# Circulating tumour cell gene expression and chemosensitivity analyses: predictive accuracy for response to multidisciplinary treatment of patients with unresectable refractory recurrent rectal cancer or unresectable refractory colorectal cancer liver metastases

**DOI:** 10.1186/s12885-022-09770-3

**Published:** 2022-06-16

**Authors:** Stefano Guadagni, Francesco Masedu, Giammaria Fiorentini, Donatella Sarti, Caterina Fiorentini, Veronica Guadagni, Panagiotis Apostolou, Ioannis Papasotiriou, Panagiotis Parsonidis, Marco Valenti, Enrico Ricevuto, Gemma Bruera, Antonietta R. Farina, Andrew R. Mackay, Marco Clementi

**Affiliations:** 1 Department of Applied Clinical and Biotechnological Sciences, University of L’Aquila, 67100 L’Aquila, Italy; 2 Department of Oncology and Hematology, Azienda Ospedaliera “Ospedali Riuniti Marche Nord”, Pesaro, Italy; 3 Department of Prevention and Sports Medicine, University Hospital Klinikum rechts der Isar, Technical University of Munich, Munich, Germany; 4 Department of Physiology and Pharmacology, Cumming School of Medicine, University of Calgary, Calgary, AB Canada; 5 Research Genetic Cancer Centre S.A, Florina, Greece; 6 Research Genetic Cancer Centre International GmbH, Zug, Switzerland

**Keywords:** Predictive accuracy, Precision oncotherapy, Liquid biopsies, Circulating tumor cells, Chemosensitivity tests, Tumor gene expressions analyses, Recurrent rectal cancer, Colorectal cancer liver metastases, Intraarterial chemotherapy, Targeted therapy

## Abstract

**Background:**

Patients with unresectable recurrent rectal cancer (RRC) or colorectal cancer (CRC) with liver metastases, refractory to at least two lines of traditional systemic therapy, may receive third line intraarterial chemotherapy (IC) and targeted therapy (TT) using drugs selected by chemosensitivity and tumor gene expression analyses of liquid biopsy-derived circulating tumor cells (CTCs).

**Methods:**

In this retrospective study, 36 patients with refractory unresectable RRC or refractory unresectable CRC liver metastases were submitted for IC and TT with agents selected by precision oncotherapy chemosensitivity assays performed on liquid biopsy-derived CTCs, transiently cultured in vitro, and by tumor gene expression in the same CTC population, as a ratio to tumor gene expression in peripheral mononuclear blood cells (PMBCs) from the same individual. The endpoint was to evaluate the predictive accuracy of a specific liquid biopsy precision oncotherapy CTC purification and in vitro culture methodology for a positive RECIST 1.1 response to the therapy selected.

**Results:**

Our analyses resulted in evaluations of 94.12% (95% CI 0.71–0.99) for sensitivity, 5.26% (95% CI 0.01–0.26) for specificity, a predictive value of 47.06% (95% CI 0.29–0.65) for a positive response, a predictive value of 50% (95% CI 0.01–0.98) for a negative response, with an overall calculated predictive accuracy of 47.22% (95% CI 0.30–0.64).

**Conclusions:**

This is the first reported estimation of predictive accuracy derived from combining chemosensitivity and tumor gene expression analyses on liquid biopsy-derived CTCs, transiently cultured in vitro which, despite limitations, represents a baseline and benchmark which we envisage will be improve upon by methodological and technological advances and future clinical trials.

## Background

Clinical and biological prognostic markers identify patients with differing risks of a specific outcome regardless of treatment, such as progression or death [1], and can select individuals at high risk of relapse, as potential candidates for alternative treatments. In contrast, predictive markers are associated with response (benefit) or lack of response to a particular therapy relative to other available therapies and identify patients more likely to benefit from a particular treatment [2]. Within this context, tissue and liquid biopsy precision oncotherapy holds great promise in improving therapeutic outcomes, as both represent important methods for detecting prognostic and predictive markers, with additional potential to identify therapeutic targets. This is particularly true for less invasive, serially repeatable liquid biopsies, which are potentially more relevant to disseminated disease.

Tissue biopsies, in accordance with American Society of Clinical Oncology (ASCO) and European Society for Medical Oncology (ESMO) recommendations [3], can be used to detect oncogenic mutations, oncogene overexpression and to evaluate chemosensitivity [4]. Liquid biopsies (blood, ascites, urine, pleural effusion, or cerebrospinal fluid) can be used to do the same but also permit purification and analysis of tumour-derived components including circulating tumor cells (CTCs), exosomes, circulating tumor DNAs (ctDNA), microRNAs (miRNAs), long non-coding RNAs (lncRNAs) and proteins [5, 6], with enhanced potential for identifying prognostic markers, predictive markers and therapeutic targets.

When compared to tumor-derived non-cellular components, intact CTCs not only provide a valuable source of quality tumour cell-derived nucleic acids and proteins for immunocytological, fluorescence in situ hybridization (FISH), DNA sequence, PCR, RT-PCR and multiplex RNA analysis but also the opportunity for transient in vitro culture, enabling chemosensitivity and tumor gene expression analysis to be performed on the same CTC population. Up to 2010, CTC detection and purification methodologies were based upon either biophysical (i.e. Biocoll/Ficoll®, Oncoquick® or Screen-Cell® Cyto IS - Sarcelles, France) or antigenic (i.e. CellSearch® system - Menarini Silicon Biosystems) properties. Following 2010, progress in microfluidic-based technologies, devices and principles have resulted in the use of novel antibody-based marker-dependent platforms, physical characteristic-based platforms, and secretome and transcriptome-based platforms, the latter of which, however, involves CTC lysis, eliminating the possibility of CTC culture for drug screening.

The FDA has approved liquid biopsy [7] and has validated the Cell-SearchTM system (Veridex LCC, Raritan, NJ, USA) as a diagnostic tool for CTC identification and enumeration in a variety of tumours, including colorectal cancer (CRC) and currently holds a dominant position amongst competitors [8]. This system is employed to detect CD45 (Cluster of differentiation 45) negative, EpCAM (Epithelial adhesion molecule) and cytokeratins CK8/18/19 positive CTCs, using anti human CK8/18/19/20 antibodies conjugated with ferrous beads for magnetic purification [8]. Cytokeratins and particularly CK20, are well-established markers of epithelial cell and metastatic CRC CTCs [9] and are employed also for semi-quantitative qRT-PCR analyses [10] and the majority of studies using this system report CTC enumeration as a prognostic marker for progression free survival (PFS) and overall survival (OS) [11]. Drawbacks with this approach, however, include potential CTC underestimation due to epithelial to mesenchymal transition (EMT) and loss of epithelial marker expression, technical bottlenecks and cost, which reduce routine use [8]. Alternative isolation and analytical methods and techniques have also been reported that overcome some of these problems (i.e. leukapheresis and Hydro-Seq technology) [8] and flow cytometry has also been reported to detect CTCs with high sensitivity and specificity [12].

To date, few papers have investigated the therapeutic predictive potential of CTC isolation and enumeration in CRC patients [13–16] or the promise of combining molecular profiling with chemosensitivity analysis, considered for some time to be the best way to improve disease characterisation and selection of individualised therapy [17, 18]. Within this context, patients presenting with unresectable recurrent rectal cancer (RRC) or unresectable CRC liver metastases, refractory to at least two lines of standard systemic chemotherapy, targeted and radiation therapy, represent good potential candidates for determining the predictive accuracy of combined in vitro (ex-vivo) CTC chemosensitivity and gene expression analysis.

CRC is currently the third most common cancer type, with upwards of ≈ 1.8 million new cases and ≈ 9% of all cancer-related deaths reported annually worldwide [19]. Treatment strategies depend upon disease stage, patient condition, molecular mechanisms, economic parameters, health care systems and unexpected factors, such as the current Sars-Covid-2 pandemic. In highly developed countries, 5-year survival rates for localized CRC of ≈ 90% plummet to ≈ 14% for metastatic or relapsed disease. For stage IV metastatic or recurrent CRC, devoid of known cancer driving mutations or targetable biomarkers (≈ 85% of patients), current systemic therapeutic regimes include: FOLFOX (leucovorin plus 5-fluorouracil plus oxaliplatin), CAPEOX (capecitabine plus oxaliplatin), FOLFIRI (leucovorin plus 5-fluorouracil plus irinotecan), FOLFOXIRI (leucovorin plus 5-fluorouracil plus oxaliplatin plus irinotecan), typically combined with bevacizumab or cetuximab or aflibercept and recently new oral drugs such as regorafenib and trifluridine/tipiracil [18, 20–28]. Patients with dominant liver metastatic disease or limited extrahepatic disease, may receive liver-directed intraarterial therapies such as hepatic arterial chemotherapy infusion, chemoembolization [20] and radioembolization to improve local tumor response and to reduce systemic side effects [18]. For all of these patients, liquid biopsy-derived CTCs deserve thorough investigation as a potential source of important predictive information with respect chemosensitivity and chemoresistance, enhancing the possibility of identifying novel alternative therapeutic strategies and reducing toxicity.

For stage IV metastatic or recurrent CRCs refractory to standard systemic therapies, that exhibit detectable overexpression or mutation of known driver oncogenes (≈ 4–15%), precision oncotherapy represents a viable therapeutic option, in conditions of: i) deficient mismatch repair protein expression (dMMR) and/or DNA microsatellite instability (MSI-H/high), treatable with pembrolizumab and nivolumab or a combination of nivolumab and ipilimumab check-point inhibitors in first and subsequent lines of treatment; ii) specific BRAF^V600E^ mutation treatable with a combination of encorafenib and cetuximab in second or third lines; iii) EGFR overexpression treatable with cetuximab and panitumumab; iv) HER-2 3+ overexpression or HER2 FISH/ISH amplification treatable with trastuzumab, lapatinib, tucatinib and deruxtecan/trastuzumab; v) neurotrophic tropomyosin receptor kinases (NTRKs) overexpression or expression of novel NTRK chimeric fusions treatable with larotrectinib or entrectinib [21–28].

Within this context, we report a retrospective study of the accuracy of in vitro ex vivo CTC chemosensitivity and tumour gene expression analyses in predicting response to treatment in CRC patients presenting with unresectable refractory RRC or unresectable refractory CRC liver metastases. In this setting, chemotherapeutic agents and monoclonal antibodies used for the multidisciplinary treatment of patients with refractory CRC, were chosen from chemosensitivity assays performed on transiently in vitro cultured liquid biopsy-derived CTCs and from the ratio of tumor gene expression exhibited by the same CTC populations compared to purified peripheral blood mononuclear cells from the same patient.

## Methods

### Patients

This study involved patients with unresectable and predictable disease course and was approved by ASL n.1 Ethics committee, Abruzzo, Italy (Chairperson: G. Piccioli; protocol number 10/CE/2018; approved: 19 July 2018 (n.1419)]. Drug selection, clinical treatments and evaluations were performed at the University of L’Aquila, L’Aquila, Italy. CTC isolation, culture, gene expression and chemosensitivity analyses were performed at the Research Genetic Cancer Centre, Florina, Greece. All patients provided written consent and received complete information about their disease and the implications of the proposed conventional treatment, in accordance with the Helsinki Declaration and the University of L’Aquila committee on human experimentation.

From a cohort of 168 CRC patients, enrolled from 2007 to 2019, comprising 62 patients with unresectable recurrent rectal cancer in progression following two lines of systemic chemotherapy and radiotherapy and 106 patients with unresectable CRC liver metastases in progression after two lines of systemic chemotherapy, 36 patients were retrospectively selected based on the following criteria: submission for precision oncotherapy by combined intraarterial chemotherapy and systemic venous targeted therapy, using drugs selected by comparative chemosensitivity and gene expression analyses performed on in vitro cultured CTCs and PBMCs from the same patient. To ensure the stability and consistency of sample collection and detection, only patients submitted for combined locoregional intraarterial chemotherapy and systemic venous targeted-therapy, whose CTCs had been purified using the same methodology, were included in this retrospective study, whereas patients submitted for systemic venous chemotherapy and/or systemic targeted therapy, and those in which CTC isolation and culture had been performed with more recent techniques and methodologies, not available at the beginning of the study, were excluded.

Decisions concerning unresectability and precision oncotherapy were made by experienced surgeons, oncologists and radiologists during multidisciplinary meetings. Inclusion criteria were: i) histologically confirmed colorectal cancer diagnosis and complete primary tumor resection; ii) failure of two lines of systemic chemotherapy; iii) Eastern Cooperative Oncology Group (ECOG) performance status of < 4; iv) adequate liver or renal dysfunction (total bilirubin serum levels < 3 mg/dL, serum albumin level > 20 g/L, serum creatinine level < 2 mg/dL). In all cases, systemic chemotherapy ceased 4 weeks prior to the 1st cycle of tailored intraarterial chemotherapy in association with targeted therapy. Patients with inadequate medical records were excluded from this study. Demographic and clinical characteristics of the 36 patients are presented in Table 1.

**Table 1:** Patient demographic and clinical characteristics

	Median; [IQR]	Number	(%)
**Age (years)**	60.5; [55.5–67.5]		
- < 75		35	(97.2)
- ≥ 75		1	(2.8)
**Gender**			
- M		22	(61.1)
- F		14	(38.9)
**Site of recurrence/metastases**			
- Pelvis		27	(75)
- Liver		9	(25)
**Presence of other metastases**			
- Yes		18	(50)
- No		18	(50)
**Number of previous systemic chemotherapy lines**			
- 2		35	(97.2)
- > 2		1	(2.8)
**KRAS, NRAS, BRAF genotype status**			
- KRAS exon 2 codon 12		3	(8.3)
- NRAS mutations		0	(0)
- BRAF mutations		0	(0)
- Unknown		13	(36.1)
**SBTT (months)**	16.0; [12–20.5]		
**ECOG**			
- 0		0	(0)
- 1		9	(25)
- 2		13	(36.1)
- 3		14	(38.9)
- 4		0	(0)
**PFSTT (months)**	7.5; [5–12.5]		
**OSTT (months)**	34.0; [23.5–41.5]		

*IQR* Interquartile range, *SBTT* Survival from recurrence/metastases date and first treatment of tailored intraarterial chemotherapy in association with targeted therapy, *ECOG* Eastern Cooperative Oncology Group performance status before first treatment of tailored intraarterial chemotherapy in association with targeted therapy, *PFSTT* Progression free survival after tailored therapy, *OSTT* Overall survival after tailored therapy, *KRAS* Kirsten rat sarcoma virus gene, *NRAS* Neuroblastoma RAS gene, *BRAF* v-Raf murine sarcoma viral oncogene homolog B

### Liquid biopsy, CTC gene expression and chemosensitivity assays

#### Sample collection, storage and transportation

Liquid biopsies (≥ 20 mL venous blood) were collected from each patient in sterile 50 mL Falcon tubes, containing 7 ml of 0.02 M ethylenediaminetetraacetic acid (EDTA) anticoagulant (E0511.0250, Duchefa Biochemie B.V., Haarlem, The Netherlands), transported in impact-resistant containers, under refrigeration at 2–8 °C, and analysed within 80 hours [29].

#### CTC isolation

For CTC isolation, blood samples were layered over 4 ml polysucrose solution (Biocoll 1077, Biochrom, Berlin, Germany) and centrifuged for 20 min at 2500×g. Peripheral blood mononuclear cells (PBMCs) and CTCs (the buffy coat) were collected and washed with phosphate-buffered saline (PBS, P3813, Sigma-Aldrich, Germany). Cell pellets were resuspended for 10 min in erythrocyte lysis buffer [154 mM NH_4_Cl (31,107, Sigma-Aldrich, Germany), 10 mM KHCO_3_ (4854, Merck, Germany) and 0.1 mM EDTA]. Cells were then collected by centrifugation, washed in PBS, and incubated with mouse monoclonal anti-human CD45 antibody-conjugated magnetic beads (39-CD45–250, Gentaur, Belgium) for 30 min at 4 °C. Anti CD45 bead-bound cells were collected in a magnetic field and saved for use as non-cancer control PBMCs in qRT-PCR gene expression analyses. Remaining cells were incubated with mouse monoclonal Anti-Pan cytokeratin-conjugated microbeads (PCK/CK4, CK5, CK6, CK8, CK10, CK13 and CK18) (MA1081-M, Gentaur, Belgium) for 30 min at 4 °C and PCK bead-bound cells (CTCs) collected in a magnetic field and washed in PBS. Isolated viable CTCs (IV-CTCs) were counted, and samples containing ≥5 viable CTCs per ml of blood were cultured for 6 days in order to obtain sufficient cell numbers for gene expression and chemosensitivity assays.

#### CTC culture

Purified pan-cytokeratin positive/CD45-negative bead-bound cells were cultured in RPMI-1640 (R0883, Sigma-Aldrich, Germany) containing 10% Fetal Bovine Serum-FBS (F4135, Sigma-Aldrich, Germany) and 2 mmol/l glutamine (G5792; Sigma), in 12-well cell culture plates (Corning, Merck, Germany), without antibiotics, at 37 °C, 5% CO_2_, and culture medium was changed every 2 days, as previously described [30].

#### CTC validation post-culture

**Fig. 1:**
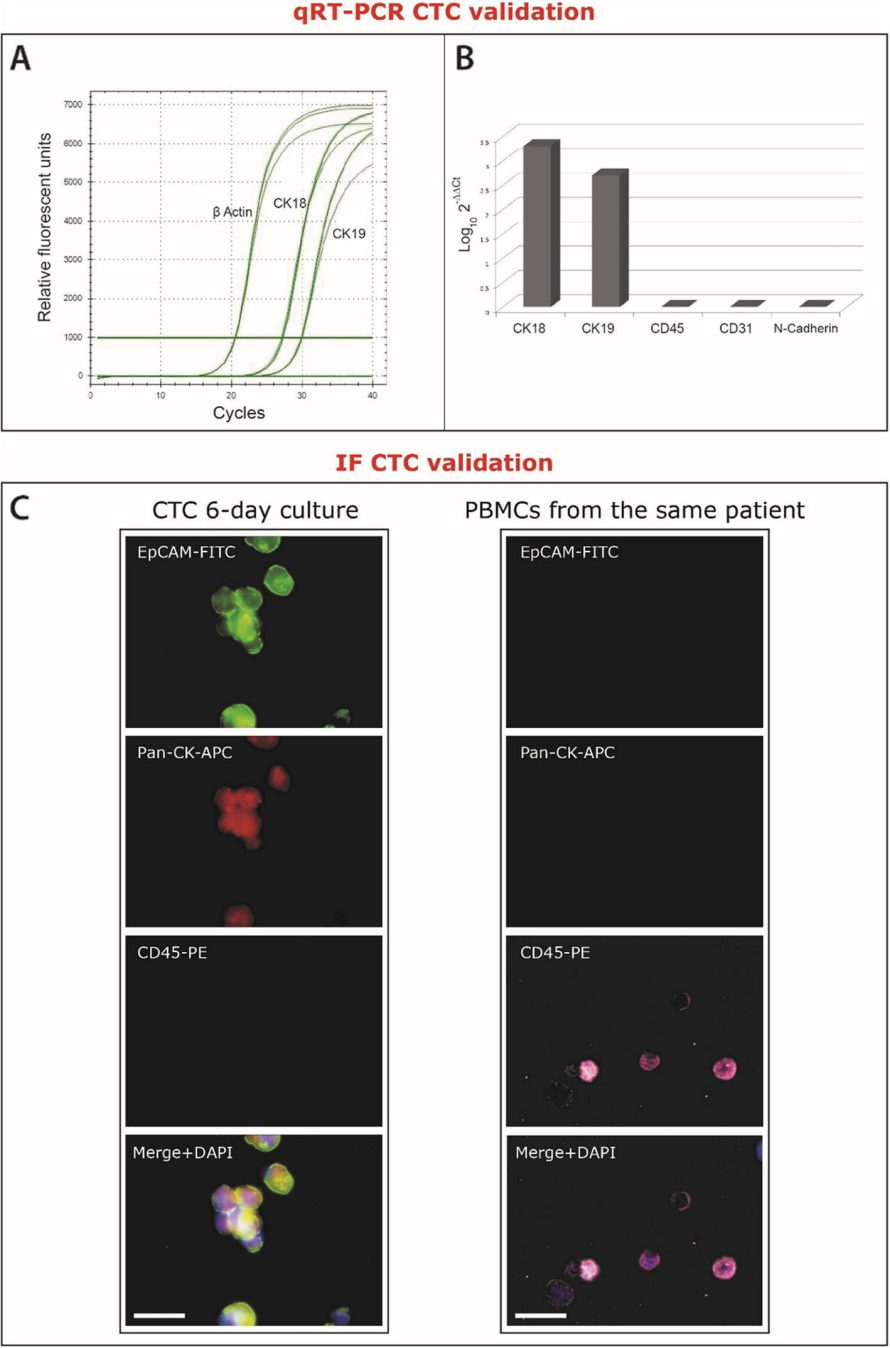
Representative: **A**) qRT-PCR CTC validation and **B**) accompanying histogram, demonstrating β-actin, CK18 and CK19 but not CD45, CD31 or N-Cadherin mRNA expression in a 6-day CTC culture, plus **C**) IF validation demonstrating positive IF immunoreactivity for EpCAM, Pan-CK but not CD45 in a 6 day CTC culture (left panels), and positive IF immunoreactivity for CD45 but not EpCAM or Pan-CK in PBMCs from the same patient (right panels) (bar = 50 μm)

CTC validation was confirmed by positive CK18, CK19, and negative CD45, CD31, N-Cadherin qRT-PCR (see section 2.2.5 for methodology) and by positive Pan-Cytokeratin-APC, and EpCAM-FITC, and negative CD45-PE Immunofluorescence (IF), in 6-day CTC cultures (Fig. 1). Briefly for IF, purified CTCs and PBMCs from the same patient were initially stained with anti-human CD45-PE (phycoerythin) conjugated mouse monoclonal antibody (304,008, Biolegend, CA, USA) and anti-human EpCAM-FITC (fluorescein isothioyanate) conjugated mouse monoclonal antibody (324,204, Biolegend, CA, USA), at recommended dilutions, and subsequently, cells were stained with anti-human Pan-Cytokeratin-APC (allophycocyanin, ab201807, Cambridge, UK) conjugated mouse monoclonal antibody (SAB4700666, Sigma-Aldrich, MO, USA), using Leucoperm staining protocol (BUF09C, Bio-Rad, CA, USA). Nuclei were counterstained with DAPI (4′,6-diamidino-2-phenylindole, Abbott Molecular, Illinois, USA) and cells were visualised under a Nikon Eclipse 50i microscope, armed with Cytovision software (Leica Biosystems, United States).

#### CTC gene expression

For gene expression analysis, RNAs were purified from CTC cultures and from corresponding PBMCs using RNeasy Mini Kits, as directed by the manufacturer (74,105, Qiagen, Hilden, Germany). RNAs (1 μg) were reverse transcribed using a PrimeScript RT Reagent Kit, as directed by the manufacturer (RR037A, Takara, Beijing, China) and subjected to KAPA SYBR Fast Master Mix (2×) Universal (KK4618, KAPA Biosystems, MA, USA) real-time qPCR, in a final volume of 20 μl. Real-time qRT-PCR reactions were performed in a final volume of 20 μl and characterised by 2 min denaturation at 95 °C, followed by 40 cycles consisting of 10 sec denaturation at 95 °C and 30 sec annealing at 59 °C. Melting-curve analysis was performed from 70 °C to 90 °C, with 0.5 °C increments of 5 s, at each step. Reactions were employed to evaluate the expression of multidrug resistance gene-ABCB1 (MDR1), thymidylate synthase (TYMS), dihydro-folate reductase (DHFR), DNA excision repair protein (ERCC1), glutathione S-transferase (GST), epidermal growth factor (EGF), vascular epidermal growth factor (VEGF), 18S ribosomal RNA (18S rRNA), β actin (ACTB), glyceraldehyde 3-phosphate dehydrogenase (GAPDH), the specific primers for which have been previously reported [31]. All reactions were performed in triplicate, compared to template-free negative controls, and analysed by Livak relative quantification [32]. Gene expression was quantified using the following equations:

$${\Delta \mathrm{Ct}}_{\left(\mathrm{threshold}\ \mathrm{Cycle}\right)}={\mathrm{Ct}}_{\mathrm{target}}-{\mathrm{Ct}}_{\beta \hbox{-} \mathrm{actin}}$$

$$\Delta \Delta \mathrm{Ct}={\Delta \mathrm{Ct}}_{\left(\mathrm{treated}\ \mathrm{CTCs}\right)}\hbox{-} {\Delta \mathrm{Ct}}_{\left(\mathrm{non}\hbox{-} \mathrm{cancer}\ \mathrm{cells}\right)}$$

$$\mathrm{Relative}\ \mathrm{expression}\ \mathrm{level}={2}^{\hbox{-} \Delta \Delta \mathrm{Ct}}$$

$$\%\mathrm{Gene}\ \mathrm{expression}=100\times \left({2}^{\hbox{-} \Delta \Delta \mathrm{Ct}}\hbox{-} 1\right)$$

and classified as low (< 50%) or high (> 50%) over-expression, as previously described [31].

#### CTC chemosensitivity

**Fig. 2:**
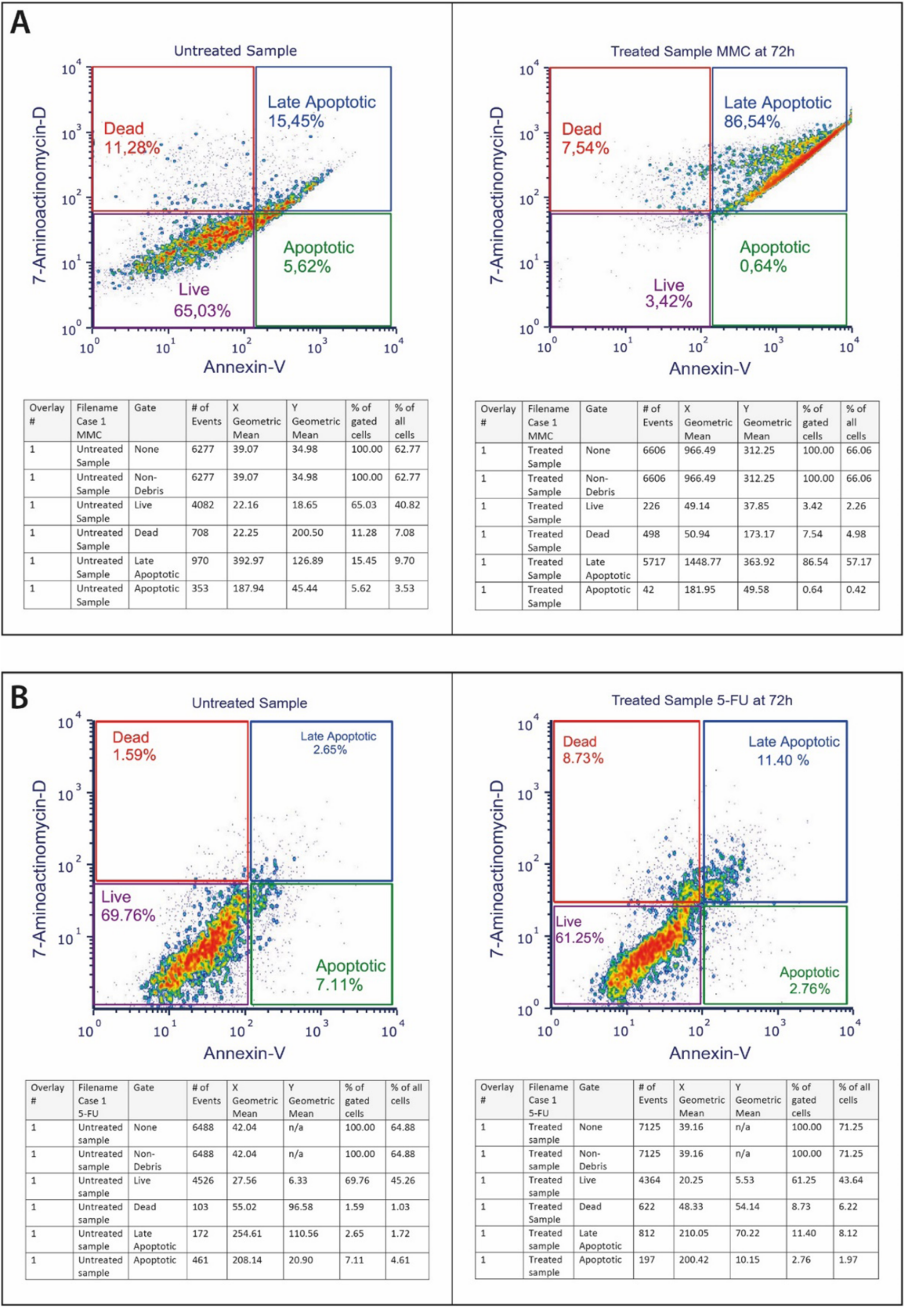
Representative flow cytometric analyses, with corresponding tables, showing the percentage changes in live, dead, late apoptotic and apoptotic Annexin-V positive CTCs, in 72 hour-chemosensitivity assays of cultured CTCs from the same individual (Case 1), demonstrating chemosensitivity to Mitomycin-C (MMC, 2 μM) (**A**) but not Flurouracil (5-FU, 10 μM) (**B**), compared to respective untreated CTC controls

For chemosensitivity assays, 6 day CTC cultures in 12-well plates were treated with the following drug concentrations: 1 μM alkeran (Μ2011, Sigma-Aldrich, Germany), 1 μM doxorubicin (D1515, Sigma-Aldrich, Germany), 1 μM cisplatin (P4394, Sigma-Aldrich, Germany), 10 μM 5-fluorouracil (F6627, Sigma-Aldrich, Germany), 1.12 μM oxaliplatin (O9512, Sigma-Aldrich, Germany), 1 μM carboplatin (41575–94-4, Sigma-Aldrich, Germany), 5 μM irinotecan (I1406, Sigma-Aldrich, Germany), 1 μM raltitrexed (112887–68-0, Sigma-Aldrich, Germany) and 2 μM mitomycin C (M4287, Sigma-Aldrich, Germany), in complete culture medium. Cell viability was assessed by Annexin V-PE (559,763; BD Bioscience, USA) flow cytometry (BD Instruments Inc., San José, CA, USA), and the percentage of living, dead and dying cells evaluated using BD CellQuest Software (BD Instruments Inc., USA (Fig. 2). Controls included untreated cells incubated in the presence and absence of chemotherapeutic drugs and cell-free counterparts. The percentage of non-viable CTCs was calculated under non-drug and drug-treated conditions, and chemosensitivity was classified as either: i) non-sensitive < 35% death; ii) partially sensitive 35–80% death, or iii) highly sensitive > 80% death.

### Precision oncotherapy protocol criteria

Decisions concerning precision oncotherapy were made by experienced oncologists, surgeons and radiologists during multidisciplinary meetings based on gene expression and chemosensitivity analyses, previous systemic chemotherapeutic protocols, and in vitro drug cytotoxicity under hypoxic conditions [33]. For intraarterial chemotherapy, drug regimens were selected according to the following criteria: i) mono-chemotherapy for CTCs with high sensitivity (> 80% of dead and dying cells) for one or more drug, with highest chemosensitivity indicating the drug to be used, ii) poly-chemotherapy for CTCs exhibiting partial sensitivity, and iii) with respect to drug activity under conditions of hypoxia. For targeted therapy, the presence/absence of KRAS and NRAS mutations in exon 2 (codons 12 and 13), exon 3 (codons 59 and 61) and/or exon 4 (codons 117 and 146) was verified, and subsequent drug selection was based upon existing therapeutic options already recommended by evolving international guidelines for clinical practice, and high CTC: PBMC gene over-expression ratios, with the highest percentage used to select each drug. Specifically, for EGFR overexpression cetuximab was selected and for high VEGFR overexpression bevacizumab was selected. If CTC: PBMC expression ratios were > 50%, target-therapy selection was based upon the highest ratio of tumour gene overexpression.

### Intraarterial chemotherapy techniques

Intraarterial chemotherapeutic procedures for unresectable refractory RRC and for unresectable refractory CRC liver metastases, together with eligibility criteria, have been described previously [17, 18]. For pelvic recurrences, hypoxic pelvic perfusion (HPP) requires specialized surgical skill and can also be performed percutaneously [34], whereas regional intraarterial chemotherapy for CRC liver metastases requires an interventional radiologist [18, 35]. Both procedures included hemofiltration to reduce toxicity [17, 18].

### Response and adverse events criteria

Responses, assessed by computed tomography (CT), magnetic resonance imaging (MRI) and positron emission tomography (PET), were evaluated 3 months following the first cycle of intraarterial chemotherapy combined with targeted therapy and classified using Response Evaluation Criteria in Solid Tumors (version 1.1), as either complete responses (CR), partial responses (PR), corresponding to a minimum 30% reduction in tumour volume, stable disease (SD) or progressive disease (PD) [36]. Patient responses prior to 2009 were re-classified retrospectively. Adverse reactions were evaluated using National Cancer Institute-Common Terminology Criteria for Adverse Events software (version 4.03) and classified from 0 to 4.

### Statistical analyses

Due to the low sample size and plausible deviations from distributional symmetry, patient demographic and clinical characteristics are summarized as percentages and median values. Cut-off values for chemosensitivity tests and tumor gene expression analyses were calculated from combined optima of positive predictive values. Specifically, this pair of cut-off values was derived from the pair of thresholds that maximized predictive accuracy, considering all possible confusion matrices. Binomial exact confidence intervals of 95% are provided for sensitivity, specificity, positive predictive, negative predictive and accuracy values.

## Results

### CTC chemosensitivity and gene expression analyses, used for selecting precision oncotherapy protocols, and patient therapeutic responses

With respect to CTC purification, the CTC detection rate in this cohort of advanced stage metastatic CRC patients was 100%, the mean (± s.e.) number of purified CTCs per millilitre of blood was 10.3 ± 0.35 (range 6.2 to 16.3) (Table 2). For 25 mls blood samples this translated into total numbers of CTCs per patient ranging from ≈ 150 to 400, which was expanded by 6-day in vitro culture to between ≈ 35,000 to 100,000 CTCs per patient for the gene expression and chemosensitivity assays used to select locoregional chemotherapy and systemic targeted therapy protocols.

**Table 2:** Protocols of intraarterial chemotherapy based on CTC chemosensitivity, and RECIST 1.1 responses

Pt	IV-CTCs	Intraarterial chemotherapy protocols			Response in association with targeted therapy
		**Drug**	**% CTC chemosensitivity**	**Dose administered**	
1R	12.2/ml	MMC	82	25 mg/m^2^	**PR**
2R	16.2/ml	5-FU	70	100 mg/m^2^	**PR**
		OX	72	80 mg/m^2^	
3R	8.4/ml	MMC	80	25 mg/m^2^	**PR**
		OX	75	80 mg/m^2^	
		RAL	70	3 mg/m^2^	
4R	12.2/ml	MMC	70	25 mg/m^2^	**PR**
		CIS	70	70 mg/m^2^	
		RAL	70	3 mg/m^2^	
5R	7.5/ml	MMC	80	25 mg/m^2^	**PR**
		RAL	70	3 mg/m^2^	
6R	6.9/ml	MMC	80	25 mg/m^2^	**PR**
		RAL	75	3 mg/m^2^	
7R	15.1/ml	ALK	70	30 mg/m^2^	**PR**
		CAR	75	100 mg/m^2^	
8R	16.3/ml	ALK	75	30 mg/m^2^	**PR**
		CAR	75	100 mg/m^2^	
9R	8.9/ml	OX	70	80 mg/m^2^	**PR**
10R	10/ml	OX	70	80 mg/m^2^	**PR**
		MMC	75	25 mg/m^2^	
11R	9.5/ml	OX	70	80 mg/m^2^	**PR**
		MMC	75	25 mg/m^2^	
12R	8.9/ml	OX	75	80 mg/m^2^	**PR**
		MMC	75	25 mg/m^2^	
13R	7.5/ml	MMC	80	25 mg/m^2^	**PR**
14R	8.9/ml	MMC	82	25 mg/m^2^	**PR**
15R	6.9/ml	MMC	75	25 mg/m^2^	**PR**
16R	12.2/ml	MMC	82	30 mg/m^2^	**PR**
17R	16.2/ml	5-FU	60	600 mg/m^2^	**PR**
		OX	72	150 mg/m^2^	
18R	7.5/ml	OX	75	80 mg/m^2^	**SD**
		IRI	80	100 mg/m^2^	
19R	9/ml	MMC	85	25 mg/m^2^	**SD**
20R	8.4/ml	OX	85	80 mg/m^2^	**SD**
21R	9.7/ml	MMC	83	25 mg/m^2^	**SD**
22R	6.9/ml	MMC	80	25 mg/m^2^	**SD**
23R	9.8/ml	MMC	75	25 mg/m^2^	**SD**
24R	16.2/ml	DOX	72	35 mg/m^2^	**SD**
		OX	65	80 mg/m^2^	
25R	9.8/ml	MMC	70	25 mg/m^2^	**SD**
		RAL	70	3 mg/m^2^	
26R	8.9/ml	MMC	80	25 mg/m^2^	**SD**
27R	6.9/ml	MMC	80	25 mg/m^2^	**SD**
28 L	9.4/ml	MMC	85	25 mg/m^2^	**SD**
29 L	16.2/ml	MMC	80	25 mg/m^2^	**SD**
30 L	7.5/ml	OX	75	150 mg/m^2^	**SD**
		IRI	80	200 mg/m^2^	
31 L	10/ml	IRI	90	200 mg/m^2^	**SD**
32 L	16.2/ml	IRI	80	200 mg/m^2^	**SD**
33 L	7.5/ml	MMC	81	30 mg/m^2^	**SD**
34 L	9.8/ml	MMC	70	30 mg/m^2^	**SD**
35 L	9.9/ml	MMC	75	30 mg/m^2^	**SD**
		OX	70	150 mg/m^2^	
36 L	6.9/ml	MMC	80	30 mg/m^2^	**PD**

*Pt* Patient, *R* Patient with unresectable refractory recurrent rectal cancer, *L* Patient with unresectable refractory colo-rectal cancer liver metastases, *IV-CTCs* Isolated viable circulating tumor cells in patient blood prior to culture, *5-FU* 5 fluorouracil, *MMC* Mitomycin, *IRI* Irinotecan, *OX* Oxaliplatin, *RAL* Raltitrexed, *DOX* Doxorubicin, *ALK* Alkeran, *CIS* Cisplatin, *CAR* Carboplatin, *PR* Partial response, *SD* stable disease, *PD* Progressive disease

RECIST 1.1 responses to combined intraarterial chemotherapy and targeted therapy, selected by CTC chemosensitivity and gene expression assays, and the KRAS mutational status of tumors are presented in Tables 2 and 3.

**Table 3:** Targeted therapy protocols selected according to CTC: PBMC percentage gene overexpression ratios, and associated RECIST 1.1 responses

Pt	EGFR %	VEGFR %	MDR1%	TYMS %	DHFR %	ERCC1%	GST %	Protocol of targeted therapy	Response in association with intraarterial chemotherapy
1R	45	70	80	0	0	0	18	BEV	**PR**
2R	45	70	80	0	0	0	10	BEV	**PR**
3R	50	65	65	0	0	0	10	BEV	**PR**
4R	50	20	55	0	0	0	10	CET	**PR**
5R	65	50	70	0	0	0	5	CET	**PR**
6R	50	80	70	0	0	0	10	BEV	**PR**
7R	50	20	80	10	0	25	20	CET	**PR**
8R	50	20	60	0	0	0	10	CET	**PR**
9R	50	30	65	10	0	0	10	CET	**PR**
10R	70	60	70	0	0	0	0	CET	**PR**
11R	60	55	65	5	0	0	10	CET	**PR**
12R	60	55	65	5	0	0	15	CET	**PR**
13R	40	70	80	5	10	0	10	BEV	**PR**
14R	50	70	70	0	0	0	10	BEV	**PR**
15R	50	20	65	0	0	0	15	CET	**PR**
16R	65	70	80	0	0	0	18	BEV	**PR**
17R	55	70	80	0	0	0	10	BEV	**PR**
18R	60	85	70	0	0	0	10	BEV	**SD**
19R	60	55	60	0	0	0	10	CET	**SD**
20R	70	45	58	0	0	0	12	CET	**SD**
21R	10	65	65	0	0	0	20	BEV	**SD**
22R	50	80	70	0	0	0	10	BEV	**SD**
23R	60	70	70	0	0	0	10	BEV	**SD**
24R	80	35	60	0	25	0	0	CET	**SD**
25R	60	70	70	0	0	0	0	BEV	**SD**
26R	40	80	70	5	0	15	15	BEV	**SD**
27R	40	60	60	0	0	0	10	BEV	**SD**
28 L	50	70	70	10	0	0	10	BEV	**SD**
29 L	40	50	60	0	10	0	10	BEV	**SD**
30 L	60	85	70	0	0	0	10	BEV	**SD**
31*L	65	80	83	0	0	0	10	BEV	**SD**
32 L	50	80	60	0	0	0	8	BEV	**SD**
33 L	60	90	70	0	0	0	10	BEV	**SD**
34 L	60	30	70	0	0	0	10	CET	**SD**
35*L	45	55	70	0	0	0	10	BEV	**SD**
36*L	50	80	70	0	0	0	10	BEV	**PD**

*CTC* Circulating tumor cells, *PMBCs* Peripheral mononuclear blood cells, *Pt* Patient, *R* Patient with unresectable refractory recurrent rectal cancer, *L* Patient with unresectable refractory colo-rectal cancer liver metastases, *EGFR* Epidermal growth factor receptor, *VEGFR* Vascular endothelial growth factor receptor, *MDR1* Multidrug resistance gene (ABCB1 gene), *TYMS* Thymidylate synthase gene, *DHFR* Dihydrofolate reductase, *ERCC1* DNA excision repair protein, *GST* Glutathione S-transferases, *BEV* Bevacizumab (dosage administered = 5 mg/kg), *CET* Cetuximab (dosage administered = 250 mg/m^2^), *PR* Partial response, *SD* Stable disease, *PD* Progressive disease

*KRAS (Kirsten rat sarcoma virus gene) mutated in codon 12, exon 2

### Response predictive accuracy of precision oncotherapy protocols, selected by chemosensitivity and gene expression analyses performed on in vitro cultured liquid biopsy-derived CTCs and PBMCs

With respect to the 36 patients assessed in this study, intraarterial chemotherapy combined with targeted therapy elicited 17 partial responses (PR), 18 stable disease (SD) responses and 1 progressive disease (PD) response. RECIST 1.1 responses subdivided into positive (CR + PR) and negative (SD + PD) responses, in relation to CTCs chemosensitivity and tumor gene expression tests for targeted therapy, are presented in Table 4. Tests were defined as positive when ≥70% of drug-treated CTCs were killed and CTC: PBMC tumor gene expression ratios were ≥ 50%. Tests were defined as negative when < 70% of drug-treated CTCs were killed and CTC: PBMC tumor gene expression ratios were < 50%.

**Table 4:** Positive and negative RECIST 1.1 responses after intraarterial chemotherapy and targeted therapy, using protocols selected by positive or negative CTC chemosensitivity and tumor gene expression analyses

	RECIST 1.1 Response to intra-arterial chemotherapy and targeted therapy selected by CTC chemosensitivity and tumor gene expression analyses		
CTC chemosensitivity assays for intra-arterial chemotherapy and CTC tumor gene expression assays for targeted therapy	Positive patient responses (CR + PR)	Negative patient responses (SD + PD)	Total
**Positive test** [CTC death ≥70% plus Gene overexpression ≥50%]	16	18	34
**Negative test** [CTC death < 70% plus Gene overexpression < 50%]	1	1	2
**Total**	17	19	36

*CTC* Circulating tumor cell, *CR* Complete response, *PR* Partial response, *SD* Stable disease, *PD* Progressive disease

Considering both types of therapy (intraarterial and targeted) and associated respective cut-offs for assay positivity, we report 16 true positive responses (TP), 1 false positive response (FP), 18 true negative responses (TN) and 1 false negative response (FN), which translate into a sensitivity of 94.12% (16/17) (95% CI 0.71–0.99), specificity of 5.26% (1/19) (95% CI 0.01–0.26), a positive predictive value of 47.06% (16/34) (95% CI 0.29–0.65), a negative predictive value of 50% (1/2) (95% CI 0.01–0.98) and an over-all predictive accuracy of 47.22% (17/36) (95% CI 0.30–0.64).

### Adverse events

No technical, hemodynamic or vascular complications were observed during HPP and HAI procedures, no perfusion-related postoperative deaths were registered, and all adverse events are reported in Table 5.

**Table 5:** Adverse events in 27 patients with unresectable refractory RRC and 9 patients with unresectable refractory CRC liver metastases submitted for Hypoxic Pelvic Perfusion (HPP)/targeted therapy or Hepatic Artery Infusion/targeted therapy based on in vitro CTC chemosensitivity and gene expressions analyses

Adverse events		HPP/target-therapy, *N* = 27	HAI/target therapy, *N* = 9	Total, *N* = 36
	Grade	N (%)	N (%)	N (%)
Bone marrow hypocellurarity	−1/2	3 (11)	1 (11)	4 (11)
	−3/4	1 (4)	0	1 (3)
Nausea/vomiting	−1/2	3 (11)	1 (11)	4 (11)
	−3/4	0	1 (11)	1 (3)
Diarrhea	−1/2	0	1 (11)	1 (3)
	−3/4	0	0	0
Alopecia	−1/2	2 (7)	1 (11)	3 (8)
	−3/4	0	0	0
Fatigue	−1/2	2 (7)	1 (11)	3 (8)
	−3/4	0	0	0
Neuropaty	−1/2	2 (7)	1 (11)	3 (8)
	−3/4	0	0	0
Hand Foot Syndrome	−1/2	0	1 (11)	1 (3)
	−3/4	0	0	0
Rash	−1/2	0	2 (22)	2 (5.5)
	−3/4	0	0	0
Non-infectious fever	−1/2	0	4 (44)	4 (11)
	−3/4	0	0	0
Perineal skin toxicity	−1/2	6 (22)	0	6 (16.6)
	−3/4	5 (18)	0	5 (14)
AST and ALT increased	−1/2	0	1 (11)	1 (3)
	−3/4	0	0	0
Persistent leakage of fluid from the inguinal incision	−1/2	3 (11)	0	3 (8)
	−3/4	1 (4)	0	1 (3)
Inguinal hematoma	-1/2	1 (4)	1 (11)	2 (5.5)
	-3/4	0	0	0

*AST* Aspartate transaminase, *ALT* Alanine transaminase

## Discussion

Interest in precision oncotherapy has grown dramatically over the past 20 years and routine characterisation of oncogenic pathways activated in primary, recurrent and metastatic tumour tissue biopsies form the mainstay of precision oncology selection of individualised therapeutic strategies, which has led to improvements in survival rates. Tissue biopsy procedures, however, are invasive, can facilitate tumour dissemination and are of limited use in patient follow-up. These drawbacks have stimulated interest into the potential use of liquid biopsies, as a source of tumour-derived components including CTCs that can be enumerated as a prognostic marker, purified and characterised at the biomolecular and chemosensitivity levels to provide predictive information for precision oncology.

Although current clinical use of liquid biopsies remains limited, CTCs have been isolated from patients with a variety of tumour types [37, 38], including CRC [17, 18]. CTC research has evolved from prognostic studies based upon CTC enumeration, to predictive clinical response studies based upon the in vitro chemosensitivity and tumor gene expression profiles of cultured CTCs. Various methods for CTC isolation and enumeration have been reported [10], although FDA approval of a particular method and equipment has led to a commanding position [8]. Furthermore, in addition to liquid biopsy-derived CTC purification methodologies, a variety of methods have been reported for subsequent CTC in vitro cultivation in order to facilitate chemosensitivity and tumor gene expression analysis [12, 39].

A potential drawback of current methodologies, however, is the exclusive use of epithelial markers for detecting and purifying CTCs that may miss CTCs that have undergone epithelial to mesenchymal transition (EMT). EMT is a pre-requisite for metastatic dissemination in majority of carcinomas, is characterised by a shift to SNAIL, ZEB and TWIST transcriptional activity, TGFβ and Wnt signaling and matrix metalloproteinase activity and results in repression of epithelial marker expression and “metaplastic” conversion to a motile mesenchymal phenotype [10]. CTCs numbers may, therefore, be underestimated by methods that exclusively employ epithelial markers. Furthermore, purified epithelial CTCs may differ to mesenchymal CTCs in biomolecular profiles and chemosensitivity, potentially making information from CTC analysis incomplete, more relevant to epithelial rather than mesenchymal tumour components and may differ to information gained from tissue biopsies, leading to differences in precision oncotherapy predictions of clinical efficacy between liquid and tissue biopsies.

Given these variables and considerations, the salient question remains whether it is possible to provide a calculated prediction of clinical response to select tailored drugs using a specific CTC methodology, and would this alter if the tumour type, patient population or analytical methodology changes?

The predictive accuracy of precision oncotherapy depends initially upon patient characteristics, tumor type and stage. Here, we evaluated the predictive accuracy of precision oncotherapy on a specific population of CRC patients, refractory to at least two standard lines of systemic therapy, undoubtedly more chemo-resistant than patients with unresectable recurrent rectal cancer or unresectable CRC liver metastases submitted to first line treatment. The predictive accuracy of precision oncotherapy also depends upon the methodology used for CTC isolation, enrichment, in vitro culture, chemosensitivity, tumor gene expression, etc. In this study, due to the long accrual period, the predictive accuracy of a particular methodology for CTC purification, culture and analysis, was evaluated. This methodology has now been improved by recent technical advances, which if available during the period of study, may have enhanced predictive accuracy.

Based on a predictive accuracy value of 47.2% for the methodology employed, the main clinical message is that patients with unresectable recurrent rectal cancer or unresectable CRC liver metastases, refractory to at least two lines of traditional systemic therapy, exhibited a positive RECIST 1.1. response in ≈ 50% of cases. This represents the first reported estimation of predictive accuracy derived from combined chemosensitivity and tumor gene expression analysis of in vitro cultured CTCs and extends a recent meta-analysis reporting a predictive value of chemosensitivity assays alone for individualized CRC chemotherapy [40]. Furthermore, the combined positivity cut-off of ≥70% cell death for chemosensitivity and ≥ 50% for CTC: PBMC tumor gene expression ratios, were selected considering the positive predictive value, which essentially focus on an optimized RECIST 1.1 response, maximizing predictive accuracy. Choosing pairs of lower cut-off values, potential oncotherapy precision protocols should consider chemotherapeutic agents for which CTCs exhibit greater resistance and/or monoclonal antibodies for less expressed CTC antigens.

The reported predictive accuracy value of 47.2%, is indeed impressive considering that the CTCs from 77.8% of patients exhibited over-expression of the multi-drug resistance gene MDR1 (≥ 65%), CTCs from 19.5% of patients exhibited significant over-expression of ERCC1 and GST (> 15%), involved in platinum resistance [41] and CTCs from 22.8% of patients exhibited ≥5% over-expression of TYMS or DHFR, involved in 5-fluorouracil resistance [42, 43].

Another important message from this study is that transient in vitro culture of liquid biopsy-derived CTCs can provide sufficient cell numbers for screening anticancer compounds including agents not normally prescribed for any particular tumor type, of relevance for “drug repurposing” [39]. Indeed, 72% of CTCs in the refractory CRC patient group exhibited sensitivity to Mitomycin C, 5.5% exhibited sensitivity to Alkeran and 2.7% exhibited sensitivity to Doxorubicin, agents that are not currently recognized as particularly active against CRC and have been reported to be ≈10 times more cytotoxic under hypoxic conditions [33]. In the present study, CTC chemosensitivity assays were not performed under hypoxic conditions, suggesting that the cytotoxic potential of these agents could be further enhanced via intraarterial administration to improve access to hypoxic tumour regions or using therapeutic protocols that promote tissue hypoxia, such as hypoxic pelvic perfusion (HPP), in order to take therapeutic advantage of chronic or transient tumour tissue hypoxia [44].

Limitations of this study, include: i) the small patient sample size, which in any case provided a confidence interval for predictive accuracy of 95% (CI 0.30–0.64); ii) the inclusion of data from CTC populations obtained from patients with recurrent rectal cancer and CRC liver metastases, mitigated somewhat by the need to compare CTCs disseminating from recurrent and overt metastatic sites; iii) transient in vitro CTC culture, used to obtain sufficient numbers for assay, which may have altered gene expression, chemosensitivity and reduced predictive accuracy, deemed necessary for reasons of methodological homogeneity for this study, initiated 2007 and terminated in 2019, which also explains why novel miniaturized system-based methods were not used; iv) the use of methodology for the evaluation of apoptosis that may have underestimated total death by not measuring paraptosis, ferroptosis and/or necroptosis; and v) lack of novel recent methodological improvements (microfluidic based systems, anti-CK20 antibodies and EMT markers), also for reasons of methodological homogeneity for the duration of this 2007–2019 retrospective study.

Despite these limitations and emphasizing combined treatment with intraarterial chemotherapy and systemic target therapy for advanced CRC patients, refractory to at least two lines of systemic therapy, this study provides interesting biostatistical information for multidisciplinary oncological teams for quantifying the predictive accuracy of the particular CTC-isolation/assay methodology employed. We do not, however, exclude the possibility that predictive accuracy could be improved by recent technological and biomolecular innovations (see above) nor does this study evaluate the relative importance of intraarterial chemotherapy in determining tumor response.

## Conclusions

The biomolecular methodology utilised in this retrospective study of patients with unresectable refractory RRC and unresectable refractory CRC liver metastases, provides a predictive accuracy of ≈ 50% for response to liquid biopsy precision oncology-selected intraarterial chemotherapy and targeted therapy. We envisage that this value will be improved by novel technologies and therapeutic agents, and by upgrading methodology to ensure purification of all potentially metastatic epithelial, quasi-mesenchymal and mesenchymal CTC sub-populations, to include physiologically relevant controls in addition to PBMCs, and by eventual methodological standardization. Despite the challenges in applying these procedures to wider populations in non-research settings, the encouraging result of this retrospective study, employing CTC purification procedures relevant to 2007–2019, sets the stage for potential improvements in predictive accuracy in subsequent clinical trials employing current and future technological and methodological improvements.

## Data Availability

The datasets generated and/or analysed during the current study are not publicly available due to privacy reasons but are available from the corresponding author on reasonable request.

## Abbreviations

FDA: Food and Drug Administration
BRAF: v-Raf murine sarcoma viral oncogene homolog B
CK-20: Cytokeratin-20
EGFR: Epithelial Growth Factor Receptor
EMT: Epithelial-Mesenchymal-Transition
CD45: Cluster of differentiation 45
EpCAM: Epithelial adhesion molecule
pan-CK: Pan-Cytokeratin
qRT-PCR: Reverse Transcriptase quantitative Polymerase Chain Reaction
SNAIL: Gene family of zinc-finger transcription factors
ZEB: Gene family of zinc finger transcription factors
TWIST: Twist gene family
TGFβ: Transforming growth factor β
Wnt: Wingless and Int − 1 signaling pathway
CT: Computed tomography
MRI: Magnetic resonance imaging
PET: Positron emission tomography
CD31: Platelet endothelial cell adhesion molecule
DAPI: 4′,6-diamidino-2-phenylindole
EpCAM-FITC: Antibody against Epithelial cell adhesion molecule conjugated to fluorescein isothioyanate
CD45-PE: Antibody against Cluster of differentiation 45 conjugated to phycoerythin
Pan-Cytokeratin-APC: Antibody against a panel of cytokeratins conjugated to allophycocyanin
ACTB: β actin
18S rRNA: 18S ribosomal RNA
5-FU: 5 fluorouracil
GAPDH: Glyceraldehyde 3-phosphate dehydrogenase
MMC: Mitomycin
IRI: Irinotecan
OX: Oxaliplatin
RAL: Raltitrexed
DOX: Doxorubicin
ALK: Alkeran
CIS: Cisplatin
CAR: Carboplatin
